# The application of production-oriented approach research teaching method in medical academic English course

**DOI:** 10.1371/journal.pone.0296249

**Published:** 2024-02-29

**Authors:** Zheng Liu

**Affiliations:** College of Medical Laboratory Science, Guilin Medical University, Guilin, Guangxi, China; Ahvaz Jundishapur University: Ahvaz Jondishapour University of Medical Sciences, ISLAMIC REPUBLIC OF IRAN

## Abstract

The purpose of this study is to investigate the efficiency of the production-oriented approach research (POA-R) teaching approach on academic English courses. The six-week study involved thirty-nine postgraduate students from Guilin Medical University studying medical technology. These students were randomly divided into POA-R (19 participants) and non-POA-R (20 participants) groups. The process of study in the POA-R group was divided into three stages, motivating, enabling, and assisting. The instructor gave the class a task at the motivational stage that involved taking the academic IELTS exam, writing a review article and giving an oral presentation about their research topic. At this stage, students are challenged to find relevant information searching PubMed and other literature databases. The teacher served as a facilitator of learning and would not offer information related to the tasks. During the enabling phase, students were encouraged to ask their supervisor for help and guidance. Students ask questions in class, and the instructor discusses the questions with the students and guides them to solve the questions independently. During the assessment stage, students take the academic IELTS exam, finish the review article, given an oral presentation related their research project, and complete an instructional questionnaire. The non-POA-R group was instructed by the teacher lecture method, comprising six lectures and an oral presentation in addition to the completion of a review article. The final grades of course include a review article, an oral presentation, and an academic IELTS test. The results revealed that the students in the POA-R group outperformed the non-POA-R group in terms of mean scores on the IELTS exam, oral presentation, and review article. To further support and demonstrate the advantages of the POA-R teaching approach, an instructional questionnaire using Likert scales and the attitudes of their supervisors was employed. In conclusion, the POA-R teaching approach is a highly successful strategy for enhancing postgraduate students’ academic English proficiency. It greatly enhanced the participants’ academic knowledge, learning interest, and active learning.

## 1. Introduction

### 1.1 Research significance

Academic English courses are considered as essential core courses for postgraduate students at many universities [1, 2]. In recent years, it has become compulsory in some universities. Academic English courses at various universities have slightly different goals, but they all strive to improve students’ English language abilities and academic knowledge [3, 4]. For instance, the academic English course at Wuhan University seeks to improve postgraduate students’ academic knowledge and English language proficiency [5]. The goal of the academic English course at Pavlo Tychyna Uman State Pedagogical University is to help students apply theory to further engage in research activities [6]. According to these research, academic English courses are essential for postgraduate students’ academic and language development, especially for those from non-native English-speaking nations.

### 1.2 Research gap

Academic English is a compulsory course for medical postgraduate students at Guilin Medical University. The author has been engaged in the teaching of academic English for years. In recent years, it found that the challenges for academic English instructor. Firstly, academic English course has no its teaching characteristics. In certain universities, the academic English courses cannot be distinguished from public English courses in terms of content and teaching methods. Additionally, academic English teaching did not integrate with the postgraduate students’ research projects, so it is of little help to postgraduate students’ research. This situation creates a gap between teaching and research. For this reason above, the author creates the “Production- Oriented Approach Research” (POA-R) teaching approach based on the POA teaching theory, hoping to seek a breakthrough in academic English teaching methods [7]. With this innovative approach, students are given an assignment to finish an oral report and a review of papers related to their research project. Students independently search for literature and discuss questions with instructor in class. The POA-R teaching approach links students’ research project with academic English courses, stimulating their interest in learning. Finally, it enhances students’ active learning, academic English proficiency, and academic knowledge.

### 1.3 Research design

Postgraduate education has the characteristics of learner autonomy and research centering. Our academic English course is task-driven. The characteristics of POA-R teaching approach is that integrate learning the English language knowledge with student research projects. The POA-R teaching approach is divided into three stages: motivating stages, enabling stages, and assessing stages. During the motivating stage, the teacher explains the task. It require students to complete an IELTS exam, write an English review articles and give an oral presentation related to their research project. During the enabling stages, students try to complete this task independently. The instructor offers advice and assistance in resolving issues that students have. During the assessing stage, the instructor assess the students’ task. The effectiveness of the POA-R teaching approach is further assessed using questionnaire investigation. The supervisor’s perspective on the POA-R teaching approach is taken into account. A group of students followed traditional lecture-teaching method as a control.

## 2. Literature review

### 2.1 Academic English courses

Academic English course is a compulsory course for college and postgraduate students. Academic English courses in English-speaking countries aim to assist students in comprehending academic language and preparing for professional job [8, 9]. Due to different understandings of academic English courses, some universities in non-English speaking countries focus primarily on teaching academic English grammar, while others emphasize academic English writing and reading [10, 11]. Nonetheless, the objective of academic English course is always to create a link between academics and the English language, regardless of whether the students are from English-speaking or non-speaking countries. Currently, the two main issues with academic English course are that 1) traditional teaching methods are unable to attract students’ interest in learning, and 2) academic English teaching has not relevant to students’ research projects. As a result, students find it difficult to apply their acquired academic English skills to their research project.

### 2.2 POA teaching approach

POA is a teaching theory developed at Beijing Foreign Studies University by Professor Wen and his colleagues [12]. In contrast to other language teaching approaches, the POA begins teaching with language leaning and ends with the production. Along with speaking and writing, the production also include the ability to comprehend and interpret English knowledge. According to this theory, the POA teaching process was divided into three stages: motivating, enabling, and assessing [13]. Instructor play a leading, designing, and guiding role in the entire process. The instructor assigns assignments, explains the teaching objectives and tasks during the motivational stage. Students try to finish the assignments and become aware of their shortcomings through the learning process. During the enabling stage, the instructor provides assistance while the students search for the information according to their needs. The students’ task is assessed and graded during the assessment stage. At this stage, the teacher assesses the assignments and provides comments. The central tenet of this theory is that instructor create tasks that are challenging for students first. Students then get into trouble and want to learn. Finally, the instructor explains the teaching objectives and tasks to the students, and the students gain knowledge.

The POA teaching approach has been developed over ten years to overcome the weaknesses in English instruction in higher education. In the past decade, The POA teaching approach has been put to practice by many teachers and researchers. It is a workable and efficient teaching strategy to help non-native English speakers in colleges and universities to improve their academic English skills. After reviewing the currently published POA papers, the author concludes that the current achievements of the POA teaching approach include 1) Applying the POA teaching approach to different courses such as Medical English and Business English [14, 15]; 2) Applying the POA in different cultural contexts, such as in South Korea and Hungary [16, 17]; 3) The POA teacher and material development [18].

## 3. Materials and methods

### 3.1 Research questions

While the postgraduate academic English course is important for career development and scientific research, there are some concerns regarding how it is taught.

Question 1: What effective teaching approach can stimulate postgraduate learning interest and enhance the academic English proficiency?

Question 2: What teaching approach can integrate postgraduate research project with academic English teaching?

This article will examine the effectiveness of using the POA-R teaching approach in an academic English class. The following are the objectives of this study:

1. To assess the effectiveness of the POA-R teaching approach in academic English courses.
2. To demonstrate the implement of this POA-R teaching approach in light of teaching experience.
3. To identify the challenges that applicant of POA-R teaching approach.

### 3.2 Research design outlines

**Fig 1:**
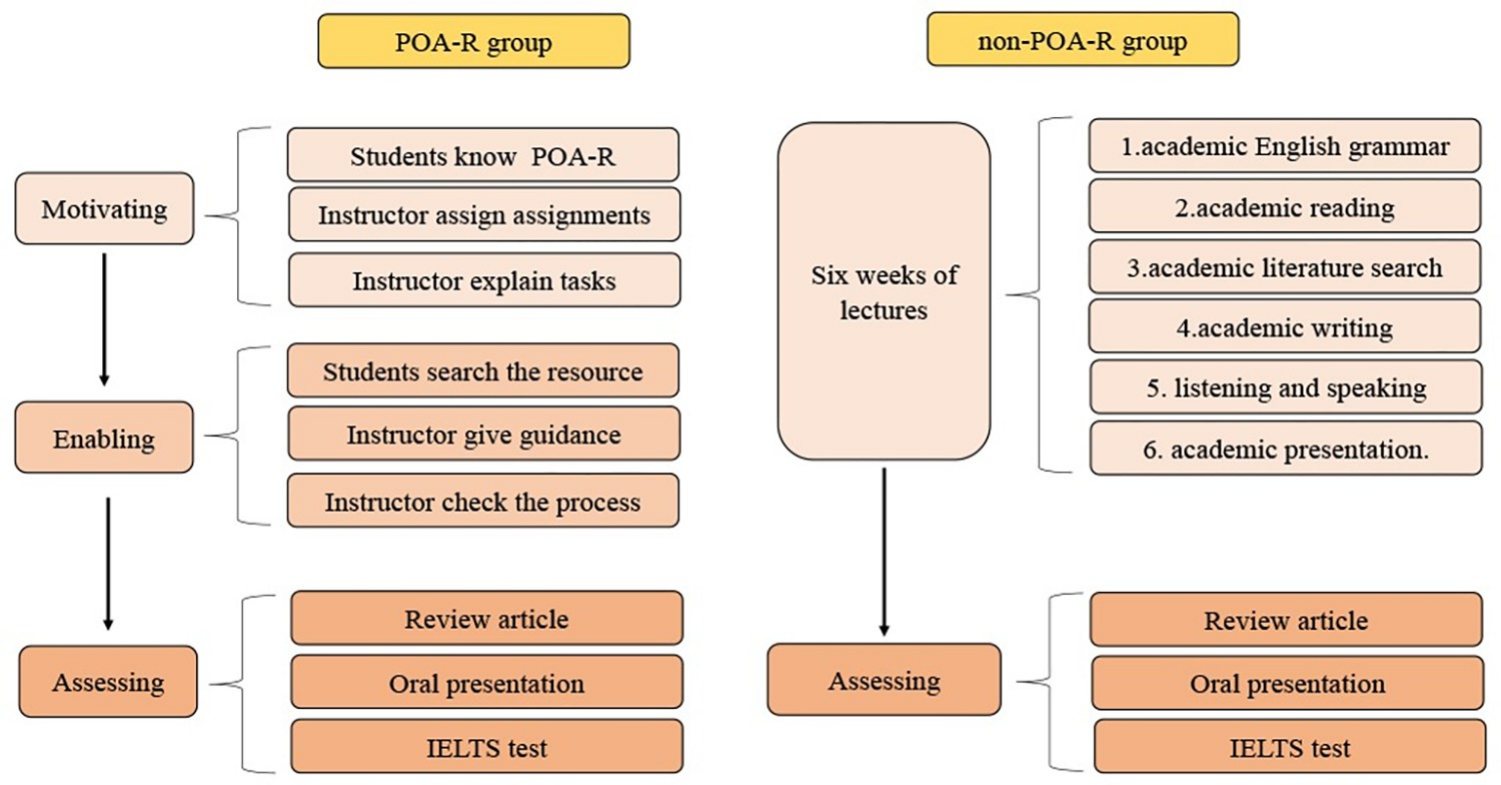
The teaching contents of POA-R and non-POA-R groups. There are differences in teaching contents between POA-R and non-POA-R groups during the 6-week academic English class for medical technology postgraduate students.

The duration of this academic English course is 6 weeks, once a week for four forty-minute sessions. A total of 39 postgraduate students majoring in medical laboratory technology participated in this course. They have been randomly divided into POA-R teaching group (19 students) and non POA-R teaching group (20 students). All students take an IELTS exam before the start of the course. Students in the POA-R group are required to talk with their supervisor in the first week to develop a review article topic. During weeks 2 to 4, POA-R students independently search for materials to finish the academic English review articles. Instructor discuss with the students and helps them work through the issue. Students are encouraged to seek help from their supervisors. In week 6, students finish an English oral presentation on the review articles. For students in the no-POA-R group, finish the review article that the lecturer assigns between weeks one through six. According to traditional teaching method, students listen to one academic English teaching topic every week. At the end of the course, all students complete an IELTS exam. The final 100 point score include IELTS scores (40%), review articles scores (30%), and oral report scores (30%). The results of the questionnaire survey and the supervisor’s assessments of the review articles were taken into consideration as references. The differences between POA-R and non-POA-R teaching approaches are depicted in Fig 1.

### 3.3 Participants

From 2022 to 2023, thirty-nine postgraduate students from Guilin Medical University’s medical technology program (13 males and 26 women; mean age, 21 years; range, 18 to 26) took part in the study. In school, they had all completed 8 to 10 years of English coursework. The two groups were randomized at random to the POA-R group (19 participants) and the non-POA-R group (20 participants). Based on the participants’ China Graduate Entrance Examination scores, the participants’ English proficiency was graded at an intermediate level. Their mother language was Mandarin. All students take the academic IELTS exam to ascertain their level of academic English because it is the standard tool used to assess students’ English proficiency levels [19]. The two English classes’ primary goals were to assist the students in developing their academic English proficiency and their capacity to successfully use English literature resources in order to finish their research projects. This is a six-week course (S1 Table). The two class students received English instruction from the same teacher for four forty-minute sessions per week. The study was approved by the ethics committee of Guilin Medical University. Informed written consent was obtained from all participants.

### 3.4 POA-R teaching design

**Fig 2:**
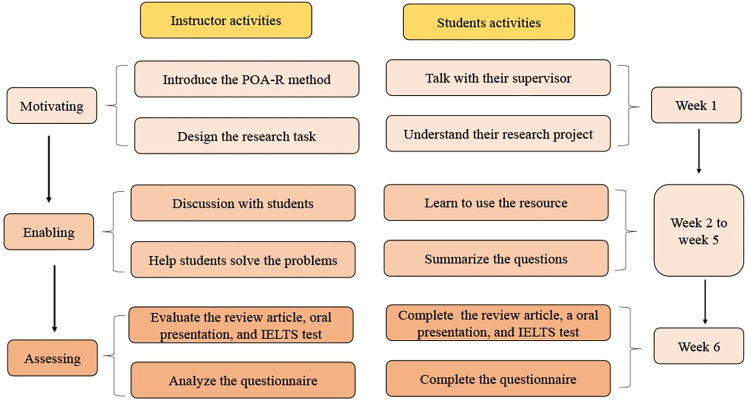
Instructor and students’ activities in the POA-R group. The actions of the instructor and students in the POA-R group during the 6-week academic English class for medical technology postgraduate students.

The POA-R group served as an experimental group. The instructor gave each student an assignment to write a research review article and create an oral presentation relevant to their research project during the first week (motivating stage) (S1 Table). The students discuss potential topics for their review articles with their supervisors. The instructor served as a facilitator of learning. During week 2 to week 5 (enabling stage), students are challenged to find useful information through PubMed and other literature databases. Each students was encourage to seek assistance and advice from their supervisor. The role of instructor in this stage is to guide students in identifying problems and assisting students in resolving those problems. Student summarized the problems and engaged in discussion. Due to the fact that the task is based on their research projects, the students are very enthusiastic about completing it. Furthermore, students report the task orally using PowerPoint slides. The instructor and other students ask questions to assist students in identifying flaws in the task, which they subsequently correct and finish. The instructor graded the oral presentation and review article during the sixth week of the course (the assessment stage). The review article also assessed by their supervisor. The academic IELTS exam is required of students as part of the assessment process. Fig 2 depicts the teacher and student activities during the POA-R teaching process.

The non-POA-R group served as the control group and was taught through traditional lecture method. During the first week of class, the lecturer gave each student a review article to write on a designed topic. Following the teaching schedule, the teacher delivered four academic papers each week that were chosen by the instructor. The instructor provided explanations of the grammar, reading, writing, speaking, listening, and presentation in the articles from the second to the fifth week (S1 Table). Students present their review article orally in the sixth week. As part of the assessment process, students must also take the academic IELTS exam. The differences in teaching content between the POA-R and no-POA-R groups can be seen from Fig 1.

### 3.5 Instructional questionnaire

A teaching approach can produce a variety of learning outcomes. An instructional questionnaire (S2 Table) was developed to determine the effectiveness of the POA-R teaching approach in academic English courses. The purpose of the questionnaire was to find out what the participants thought about how POA-R teaching approach enhance English language acquisition. It is composed of four items measuring language proficiency, two items measuring active learning, four items measuring cognitive development, and four things measuring academic knowledge. The Likert scale is used as a standard method to detect the difference between the two groups [20]. The item responses were differentiated on a 5-point Likert scale (1 strongly disagree and 5 strongly agree).

### 3.6 Data collection and analysis

The final grades includes three parts: the score of the students’ review article (30% of the total score), the score of the oral presentation (30% of the total score), and the score of the academic IELTS tests (40% of the total score). In order to ensure the reliability of the findings, the two participant groups were assessed using academic IELTS examinations at the first and sixth weeks. The oral presentation scoring criteria for students are displayed in S3 Table. S4 Table lists the scoring criteria of the review article. The data was expressed by mean± SD. Differences were considered statistically significant at *P*<0.05. All statistical analyses were performed using SPSS 18 (SPSS Inc., Chicago).

## 4. Results

### 4.1. The POA-R teaching approach effectively improve the academic English proficiency of postgraduate students

#### 4.1.1. The students in POA-R group has a higher scores in English review article

The first research question investigated whether there were any significant differences in the score of the review article between the POA-R and non-POA-R groups. Table 1 displays the mean review article scores for the two groups. Independent t-tests were employed to compare variables between these two groups. The results demonstrated that the scores on the review article differed significantly (*P* = 0.027) between POA-R and non-POA-R groups. That is, the mean score on the review article for the POA-R group (M 83.79, SD 4.42) was significantly higher than the mean score for the non-POA-R group (M 80.8, SD 3.44). Students wrote the review articles independently. This finding suggests that after using the POA-R teaching approach, students’ academic writing abilities in English much improved.

**Table 1:** The comparison of review article and oral presentation scores in the POA-R and the non-POA-R group.

	POA-R	non-POA-R	*P* value
Review article	83.79±4.42	80.8±3.44	0.027
Oral presentation	82.37±3.01	80.15±2.82	0.026

#### 4.1.2 The students in POA-R group has a higher scores in English oral presentation

The second research question examined whether there were significant differences of oral presentation between the POA-R and the non-POA-R group. The mean scores of the oral presentation for the two groups are presented in Table 1. Independent t-tests were employed to compare variables between these two groups. Between the POA-R and non-POA-R groups, there was a significant difference (*P* = 0.026) in the oral presentation scores. In other words, the non-POA-R group’s mean score (M 80.15, SD 2.82) on the review article was significantly lower than that of the POA-R group (M 82.37, SD 3.01). The outcome suggests that the POA-R teaching approach greatly enhanced the students’ oral presentation abilities.

#### 4.1.3 The students in POA-R group has a higher scores in IELTS test

The third research question examined whether the IELTS scores of the POA-R and non-POA-R groups significantly differed. Table 2 displays the mean IELTS scores for the two groups. Independent t-tests were employed to compare variables between two groups. The IELTS scores at week six showed a significant difference (*P* = 0.032) between the POA-R and non-POA-R groups. The non-POA-R group’s IELTS mean score (M 5.70, SD 0.40) was significantly lower than the POA-R group’s (M 5.89, SD 0.38). This finding suggests that participants who received instruction using the POA-R approach saw a considerable improvement in their IELTS scores. However, no significant differences were observed between the POA-R and the non-POA-R group in the first week, indicating that the IELTS level of the two groups before POA-R training is the same. Paired t-tests were used to compare variables in the groups at week 1 and week 6. The IELTS mean scores for the POA-R group and the non-POA-R group in week six were significantly higher than the mean scores from week one (*P* = 0.001). The findings indicate that following a 6-week study, the IELTS scores of the students who received lecture-teaching method also significantly improved.

**Table 2:** The comparison of academic IELTS score in the POA-R and the non-POA-R group.

	POA-R	non-POA-R	*P* value
IELTS score in the first week	5.68±0.44	5.67±0.39	0.947
IELTS score in the sixth week	5.89±0.38	5.70±0.40	0.032
*P* value	0.001	0.001	

### 4.2. POA-R teaching approach effectively integrate postgraduate research project into teaching

#### 4.2.1. Students’ views on the POA-R teaching approach

Independent-sample *t* tests were used to compare the questionnaire responses. The results showed that, for Items 1 (*P* = 0.037), Item 5 (*P* = 0.020), Item 6 (*P* = 0.013), Item 10 (*P* = 0.026), Item 11 (*P* = 0.003), and Item 13 (*P* = 0.018), there were significant differences between the two groups (Table 3). In these six items, the POA-R group’s mean score was noticeably higher than that of the non-POA-R group. The results (Item 1) showed that the POA-R teaching approach enhanced the participants’ English writing. According to the findings, the POA-R teaching approach may encourage students to engage in active learning (Items 5 and 6). Furthermore, Item 10 indicates that the POA-R teaching approach might be used in English classes in the future. In order to advance their academic understanding, it also helps students comprehend their research plan (Item 11) and synthesize the data (Item 13).

**Table 3:** The comparison for POA-R and non-POA-R groups’ six significant items.

Group	Item 1	Item 5	Item 6	Item 10	Item 11	Item 13
POA-R	4.05±0.69	4.21±0.61	4.16±0.36	4.11±0.31	4.32±0.46	4.05±0.51
non-POA-R	3.60±0.58	3.75±0.54	3.85±0.36	3.85±0.36	3.80±0.51	3.65±0.48
*P* value	0.037	0.020	0.013	0.026	0.003	0.018

#### 4.2.2 The top five ranked items in POA-R and non-POA-R group

To gain more insight into the opinions of the participants, the five items listed in Table 4 were ranked for both POA-R and non-POA-R participants according to the survey with the highest level of agreement. The order was ranked from the highest mean scores. The mean scores for the POA-R group ranged from 4.11 to 4.32. The mean scores for the non-POA-R group ranged from 3.90 to 4.30. Item 11 and Item 5 were ranked with the highest mean score (M 4.32, SD 0.46) and the second highest mean score (M 4.21, SD 0.61) of POA-R group, respectively. It suggests that the POA-R teaching approach can help students become more motivated to learn by helping them understand their research plan. It suggested that most POA-R group members focused on their research projects. According to the POA-R group, item 6 had the third-highest mean score (M 4.16, SD 0.36), suggesting that participants were motivated to learn actively by the POA-R approach. Item 8 and item 10 were ranked with the fourth and fifth highest mean scores of the POA-R group, suggesting that participants agreed the POA-R teaching approach can help them utilize the knowledge. In the future, it can be continually used in English teaching.

**Table 4:** The POA-R and non-POA-R participants’ top five ranked items.

Group	Rank	Item number/brief item statement	mean±SD
POA-R	1	Item 11/ improve the understanding of my research plan	4.32±0.46
	2	Item 5/ to enhance my learning motivation	4.21±0.61
	3	Item 6/inspires me to learn actively.	4.16±0.36
	4	Item 8/to utilize knowledge.	4.15±0.49
	5	Item 10/used in English class in future	4.11±0.31
Non- POA-R	1	Item 7/ to distinguish main ideas and details in an article	4.30±0.46
	2	Item 4/ to learn new English words	4.25±0.54
	3	Item 8/ to utilize knowledge	4.00±0.32
	4	Item 3/ to practice English speaking	3.95±0.38
	5	Item 2/ to practice English reading	3.90±0.44

In the non-POA-R group, items 7, item 4, and item 8 had the highest mean scores (M 4.30, SD 0.46), the second-highest mean scores (M 4.25, SD 0.54), and the third-highest mean scores (M 4.00, SD 0.32). It indicate that the teacher-lecture method help students understand the main ideas and details in an article, learn new vocabulary, and utilize the knowledge. The non-POA-R group participants scored item 3 (M 3.95, SD 0.38) and item 2 (M 3.90, SD 0.44) as the fourth and fifth highest mean scores, respectively. It revealed that they were in agreement that the teacher-lecture method offered efficient teaching in reading and speaking English. Based on the findings, most of the individuals in the non-POA-R group concentrated on improving their English language proficiency.

#### 4.2.3 Supervisors’ attitudes to the POA-R teaching approach

All students ‘review article were evaluated by their supervisors using the criteria listed in S4 Table. Their supervisor considered to submit 16 review articles from the POA-R group and 5 review articles from the non-POA-R group for publication. Table 5 presented the mean scores for the review article of the two groups. Independent t-tests were employed to compare variables between these two groups. The non-POA-R groups mean score (78.60±4.31) on the review article was substantially lower than that of the POA-R group (85.11±3.37). The fact that the POA-R group had higher review article scores. It suggests that the supervisor favors accepting the POA-R teaching approach. Nineteen students from the POA-R group all talked with their supervisors at least three times a month. The three most common questions are: 1) What is the main idea behind writing an academic paper? 2) How can I use the PubMed database to filter the relevant academic papers? 3) How can I incorporate concepts from other papers into mine? This indicates that the students in the POA-R group are more committed to completing the assignment. This finding suggests that students lack experience doing academic literature searches in English and incorporating the information into their review papers. The wonderful news is that two students from the POA-R group have had their review articles published in the journals Front Aging Neurosci [21] and Metabolites [22].

**Table 5:** The review article were evaluated by their supervisor.

	POA-R	non-POA-R	*P* value
Review article	85.11±3.37	78.60±4.31	0.001
Review article considered publishing	16	5	

## 5. Discussion

### 5.1 POA-R teaching approach improve participant’ academic English proficiency

This study shows that academic English language learners are greatly impacted by the POA-R teaching approach. The course design of POA-R comes from POA teaching approach. Prior studies have demonstrated the efficacy of POA teaching strategies in raising students’ English proficiency. For instance. According to Shi et al., POA is an innovative approach that is better suited for Chinese students learning English [23]. In a study, Qi et al. discovered that using POA to teach English translation can greatly increase students’ translation proficiency [24]. In academic writing and oral presentations, POA-R participants fared better than non-POA-R individuals. Within-group comparisons revealed that the POA-R participants significantly improved their IELTS scores. The POA-R participants said they had opportunities to advance their speaking, writing, and reading abilities using the POA-R teaching methodology. The questionnaire analysis produced several particularly noteworthy results that confirmed the effectiveness of the POA-R teaching strategy.

This significant English skills outcome can be attributed to the output performance designed in the POA-R class. The output performance of the POA-R teaching technique consists of an oral presentation, a discussion between the teacher and the students, and English-language academic writing. Despite the fact that the POA-R class did not hold its discussion in English, the teacher encouraged the students to speak the language by providing them with samples of English sentences. Research has shown that encouraging students to speak English in class has significantly improved their English-speaking abilities [25]. Because the participants were required to engage in English-speaking activities, they were know how POA-R teaching approach improve their academic English proficiency through language practice. For students from non-native English speaking countries, there are few opportunities for students to speak the English. Li’s study confirms what we found, which is that POA can promote the oral English learning of non-English majors [26].

### 5.2 POA-R teaching approach enhance participant’ academic knowledge

In order to complete the task, participants not only search the information through the database but also discuss the questions with the instructor and their supervisors. This activity creates a closed-loop teaching and learning process. The participant asks questions and the instructor and supervisor answer them. The participant asks new questions. It has been demonstrated that the closed-loop teaching and learning method is highly successful in helping students learning new knowledge [27]. Furthermore, there was broad acceptance of the POA-R teaching strategy for helping students learn other subjects. This study for the first time demonstrated POA-R teaching approach can improve participant’ academic knowledge. There are many teaching methods to improve students’ academic knowledge. For instance, Blázquezthe proposed that using flipped classrooms as an active learning strategy enhances students’ academic knowledge [28]. However, the teaching method of flipped classroom requires students to have a good understanding of the course content, which is very difficult for graduate students from non-English speaking countries.

The results of the survey indicated that there was broad consensus among the POA-R participants about information synthesis and data analysis. The participants were faced challenges when they analyzed an abundance of English literature information. With the assistance of their supervisor and the instructor, POA-R students were able to identify critical information from chaotic sources and synthesize relevant data. This outcome is consistent with past research showing that the POA teaching strategy helps students with data synthesis and analysis [29]. According to Shu et al., POA can assist in resolving some issues that arise during the course’s instruction [30]. As a result, I think the POA-R teaching approach offers unmatched benefits for raising students’ academic proficiency.

### 5.3 The POA-R teaching approach raises students’ motivation to study and encourages active learning

Postgraduate education is different from undergraduate education. Its goals ought to be to support students’ research projects, encourage students’ learning activities, and raise their learning interests. While traditional lecture-based teaching methods are commonly offered in universities, their failure is frequently attributed to their lack of integration with effectively learning activities [31]. Enfield stated that if the learning instruction is not interesting, it will make students not interactive in learning [32]. According to Xie’s research, using a POA-based approach to teach business English can boost students’ enthusiasm in the language [15]. POA-R teaching approach increases students’ enthusiasm for studying is further supported by the survey of POA-R participants. Our research indicates that POA-R may be the best teaching strategy for increasing postgraduate students’ enthusiasm in studying. Independent-sample t test findings from the questionnaire show that majority of the POA-R group participants concurred that this teaching method had prompted them to boost active learning. Active Learning is the process of students engaging in practices other than passively listening to lectures in order to increase their academic intake. This important finding supports Huang’s claims that the POA teaching approach can develop students into active and self-directed learners [33].

The results of the questionnaire survey showed that the POA-R teaching approach increased participants’ learning interest more than the traditional teach-lecture approach. It is evident from the activities conducted in the POA-R group that the participants have a more positive attitude toward finishing their task. For example, each participant conduct the search of the literature database to complete the review article independently. The POA-R approach can therefore simultaneously promote active learning. The students’ positive attitude during the practices helped them to significantly outperform the non-POA-R group in the article writing task. The POA English teaching approach has been shown by Lou et al. to considerably improve students’ English writing proficiency [34]. This outcome supported the idea that active learning can optimize learning outcomes [35]. Our study shows that the POA-R teaching approach significantly increases student active learning in academic English courses.

### 5.4 POA-R teaching approach is recognized by the students and their supervisors

The POA-R participants showed a very good attitude toward the POA-R teaching approach, as evidenced by the mean scores of 10 out of 14 items in the POA-R group exceeding 4. Wang’s research indicates that both teachers and students have a positive attitude towards the POA teaching approach, which is in line with the positive attitudes of POA-R participants examined in this study [36]. Additionally, the supervisors’ reflections demonstrated that they had positive opinions of the POA-R teaching approach. More review articles from the POA-R group were appreciated by the supervisor. The reason for this is that the supervisors’ interests in scientific research align with the content of the student’s review article. In addition, the student received extra credit for discussing the review article with their supervisor on time while it was being written. This phenomenon illustrates how the POA-R teaching approach helps students’ academic understanding to grow.

The supervisor was very excited about the POA-R teaching approach since, as the two students’ publications show, the class schedule and the supervisor’s research plan are tightly aligned. That’s exactly what postgraduate education calls for. Xi et al., for instance, showed that POA is very appropriate for the current modifications to college English curricula and the rising expectations placed on students’ English proficiency [37]. According to Gao et al., the POA is evaluated and shown to be useful in helping EFL students become more academically literate as they engage with authentic tasks [38]. According to Asmawi ’s research, the POA application was effective in enhancing Chinese undergraduates’ speaking skills [39]. There are many benefits to postgraduate students using the POA-R teaching approach. It links academic English study with graduate research projects. It also tackles the issue of ambiguous learning objectives in traditional academic English courses.

### 5.5. The limitations of the current study

The present research does have certain limitations. Firstly, although the students did take the IELTS exam both before and after the course, the main grades for the course were determined by their writing (English review), speaking (English oral report), and reading (read the literary). The listening scores were less involved. Future course design must take it into consideration. Second, the questionnaire survey highlighted how the POA-R teaching approach increases students’ motivation to study. However, extra methods are needed to test the improvement of students’ interest in learning in the classroom. For example, students can be asked to complete a self-assessment report and a suggestion report on the course, which can provide a comprehensive understanding of different students’ acceptance of the course. Lastly, the supervisors of the students are not as involved in the course. The supervisor’s score was not taken into account for the student’s final grade because each supervisor has distinct evaluation criteria. Courses must be further designed to allow for the supervisor to engage fully in the learning process.

## 6. Conclusion

To sum up, the POA-R teaching approach ignites postgraduate students’ passion for learning. It raises participants’ motivation to study and encourages active learning, which improves their English proficiency. Additionally, the POA-R teaching approach incorporates instruction with postgraduate research projects. Positive feedback regarding the POA-R teaching approach is widely accepted by both students and their supervisors. According to the aforementioned study findings, teaching academic English at the postgraduate level can benefit from the POA-R approach. Consequently, we recommend encouraging the use of the POA-R teaching technique in other postgraduate courses.

## Supporting information

S1 Table The class schedule in POA-R group and non-POA-R group. (DOCX)

S2 Table The 14 items included in the instructional questionnaire. (DOCX)

S3 Table Scoring criteria of oral presentation. (DOCX)

S4 Table Scoring criteria of review article. (DOCX)
