# Supplementary material for: The application of production-oriented approach research teaching method in medical academic English course
Source: PLoS One. 2024 Feb 29;19(2):e0296249. doi: 10.1371/journal.pone.0296249 (PMC10903910; doi:10.1371/journal.pone.0296249)
Supplement: S1 Table — (DOCX) [file pone.0296249.s001.docx]

**Supplementary Table 1. The class schedule in POA-R group and non-POA-R group.**

| Week | POA-R group lecture schedule | non-POA-R group lecture schedule |
| --- | --- | --- |
| 1 | Introduction of the research task | Academic English grammar |
| 2 | Discussion of literature search | Academic reading |
| 3 | Discussion of academic writing | Academic literature search |
| 4 | Discussion of academic presentation | Academic writing |
| 5 | Discussion of review article | Academic listening and speaking |
| 6 | Report the task orally | Academic presentation |
