# Supplementary material for: The application of production-oriented approach research teaching method in medical academic English course
Source: PLoS One. 2024 Feb 29;19(2):e0296249. doi: 10.1371/journal.pone.0296249 (PMC10903910; doi:10.1371/journal.pone.0296249)
Supplement: S2 Table — (DOCX) [file pone.0296249.s002.docx]

**Supplementary Table 2. The 14 items included in the instructional questionnaire.**

| Item number | Item statement | Item purpose |
| --- | --- | --- |
| 1 | I think this method helps me to practice English writing. | language proficiency |
| 2 | I think this method helps me to practice English reading. | language proficiency |
| 3 | I think this method helps me to practice English speaking. | language proficiency |
| 4 | I think this method helps me to learn new English words. | language proficiency |
| 5 | I think this method helps to enhance my learning motivation. | active learning |
| 6 | I think this method inspires me to learn actively. | active learning |
| 7 | I think this method helps me to distinguish main ideas and details in an article. | cognitive development |
| 8 | I think this method can help me to utilize knowledge. | cognitive development |
| 9 | I think this method helps me learn other subjects in future. | cognitive development |
| 10 | I think this method can be used in English class in future. | cognitive development |
| 11 | I think this method can improve the understanding of my research plan. | academic knowledge |
| 12 | I think this method can enhance my ability to analyze information. | academic knowledge |
| 13 | I think this method can enhance my ability to synthesize the information. | academic knowledge |
| 14 | I think this method can improve my presentation skill. | academic knowledge |
