# Supplementary material for: The application of production-oriented approach research teaching method in medical academic English course
Source: PLoS One. 2024 Feb 29;19(2):e0296249. doi: 10.1371/journal.pone.0296249 (PMC10903910; doi:10.1371/journal.pone.0296249)
Supplement: S3 Table — (DOCX) [file pone.0296249.s003.docx]

**Supplementary Table 3. Scoring criteria of oral presentation.**

| Evaluation part | Evaluation criterion | Score |
| --- | --- | --- |
| Presentation skills | Full of spirit and preparation | 10 |
|  | Clear organization and good timing | 10 |
|  | The intonation is loud and clear, attracting the attention of the listener | 5 |
| Explanation of article structure | Explanation of the topic | 5 |
|  | Summary and key words | 5 |
|  | Introduction, methods and references | 5 |
|  | Explanation of other parts of the article | 5 |
| Explanation of article’ results and discussion | The explanation of the results is full and complete | 10 |
|  | The discussion was explained clearly | 10 |
| question answering | Answer questions clearly | 10 |
|  | Answer the questions completely and reasonably | 10 |
| PowerPoint production | Good combination of pictures and texts | 10 |
|  | Clean, tidy and clear | 5 |
|  | **Total score** | **100** |
