# Supplementary material for: The application of production-oriented approach research teaching method in medical academic English course
Source: PLoS One. 2024 Feb 29;19(2):e0296249. doi: 10.1371/journal.pone.0296249 (PMC10903910; doi:10.1371/journal.pone.0296249)
Supplement: S4 Table — (DOCX) [file pone.0296249.s004.docx]

**Supplementary Table 4. Scoring criteria of review article.**

| Evaluation part | Evaluation criterion | Score |
| --- | --- | --- |
| Format | According to APA format | 5 |
| Grammar | Free of grammatical errors | 10 |
|  | Sentence patterns changeable | 5 |
| Content 1 | Author's name | 5 |
|  | Author's Affiliation | 5 |
|  | Abstracts within 200 words | 5 |
|  | Keywords choosing from the MeSH standard | 5 |
| Content 2 | Introduction | 10 |
|  | Main text (Reasonable structure and substantial content) | 20 |
|  | Discussion (Reasonable discussion) | 10 |
| Other sections | References (APA style) | 10 |
|  | Acknowledgments | 5 |
|  | Abbreviations list | 5 |
|  | **Total score** | **100** |
